# Assessing the efficiency and significance of Methylated DNA Immunoprecipitation (MeDIP) assays in using *in vitro *methylated genomic DNA

**DOI:** 10.1186/1756-0500-3-240

**Published:** 2010-09-16

**Authors:** Jinsong Jia, Aleksandra Pekowska, Sebastien Jaeger, Touati Benoukraf, Pierre Ferrier, Salvatore Spicuglia

**Affiliations:** 1 Centre d'Immunologie de Marseille-Luminy, Université Aix Marseille, Marseille, France; 2 CNRS, UMR6102, Marseille, France; 3 Inserm, U631, Marseille, France

## Abstract

**Background:**

DNA methylation contributes to the regulation of gene expression during development and cellular differentiation. The recently developed Methylated DNA ImmunoPrecipitation (MeDIP) assay allows a comprehensive analysis of this epigenetic mark at the genomic level in normal and disease-derived cells. However, estimating the efficiency of the MeDIP technique is difficult without previous knowledge of the methylation status of a given cell population. Attempts to circumvent this problem have involved the use of *in vitro *methylated DNA in parallel to the investigated samples. Taking advantage of this stratagem, we sought to improve the sensitivity of the approach and to assess potential biases resulting from DNA amplification and hybridization procedures using MeDIP samples.

**Findings:**

We performed MeDIP assays using *in vitro *methylated DNA, with or without previous DNA amplification, and hybridization to a human promoter array. We observed that CpG content at gene promoters indeed correlates strongly with the MeDIP signal obtained using *in vitro *methylated DNA, even when lowering significantly the amount of starting material. In analyzing MeDIP products that were subjected to whole genome amplification (WGA), we also revealed a strong bias against CpG-rich promoters during this amplification procedure, which may potentially affect the significance of the resulting data.

**Conclusion:**

We illustrate the use of *in vitro *methylated DNA to assess the efficiency and accuracy of MeDIP procedures. We report that efficient and reproducible genome-wide data can be obtained via MeDIP experiments using relatively low amount of starting genomic DNA; and emphasize for the precaution that must be taken in data analysis when an additional DNA amplification step is required.

## Background

DNA methylation at CpG dinucleotides is a major epigenetic modification with direct implications in many aspects of mammalian biology, including development and disease [1]. In normal tissues, most promoter-associated CpGs remain unmethylated, although DNA methylation does occur at promoters of a small set of genes where it generally leads to transcriptional silencing. On the other hand, cancer cells undergo dramatic changes in the level and distribution of DNA methylation [2]. Indeed, the DNA methylation-dependent silencing of many tumor suppressor genes is now recognized as a major mechanism of gene inactivation that complements genetic lesions. Recent technological advances have allowed the comprehensive analysis of DNA methylation profiles in normal and disease-associated cells [3-6]. In particular, the Methylated DNA ImmunoPrecipitation (MeDIP) assay appears to be an efficient, reproducible and cost-effective approach to characterize the methylome of large collections of DNA samples [7-10]. The overall experimental strategy is based on immunoprecipitation of methylated CpGs using a specific anti-5-methylcytidine antibody (MeDIP), as a rule followed by DNA amplification and hybridization to, typically, either CpG islands or promoter arrays. However, because the efficiency of the MeDIP assay relates to the methylated (m)CpG content and distribution within each particular genomic region [8], the quantification of DNA methylation remains approximate [11,12]. To accurately quantify CpG methylation levels, others have used *in vitro *methylated DNA in parallel to the investigated, untreated samples [e.g., [12,13]]. Here, we took advantage of this stratagem to further evaluate potential bias resulting from using MeDIP samples for DNA amplification, labeling and hybridization procedures; and also to better access the sensitivity of the overall approach.

## Results and Discussion

**Figure 1:**
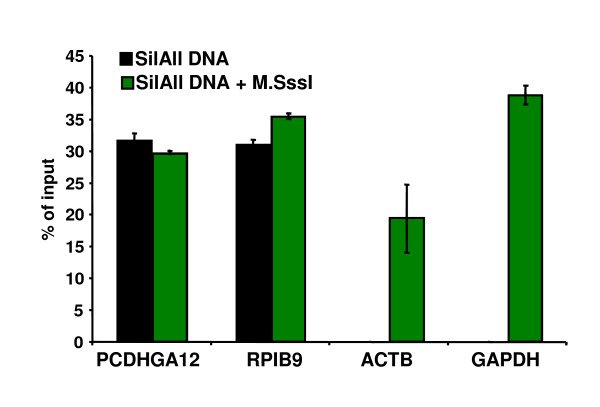
**Validation of MeDIP assay using *in vitro *methylated DNA**. DNA samples from the human cell line SilALL were methylated *in vitro *(+ M.SssI) or not, and subjected to MeDIP assay. Relative enrichment at normally methylated (PCDHGA12 and RPIB9) or unmethylated (ACTB and GAPDH) gene promoters was analyzed by real-time quantitative PCR. Both the PCDHGA12 and RPIB9 gene promoters were previously found to be commonly methylated in T-ALLs and derived cell lines [27]. Results shown represent the mean values of triplicate PCRs; and are representative of three independent experiments.

Initially, we performed MeDIP experiments using 2 μg of either untreated or M.SsssI methyltransferase-treated (i.e., *in vitro *methylated) DNA obtained from the SiLALL cancer cell line [14]. To validate the MeDIP resulting samples in terms of CpG methylation yield, we analyzed by real-time PCR the enrichment levels for several CpG-rich promoters associated with either expressed (ACTB; GAPDH) or silent (PCDHGA12; RPIB9) genes in SiLALL cells (Figure 1). As expected, these promoter regions were similarly enriched in MeDIP signals from *in vitro *methylated samples, independently of the methylation status *in vivo*, implying that *in vitro *methylation has been efficiently achieved.

**Figure 2:**
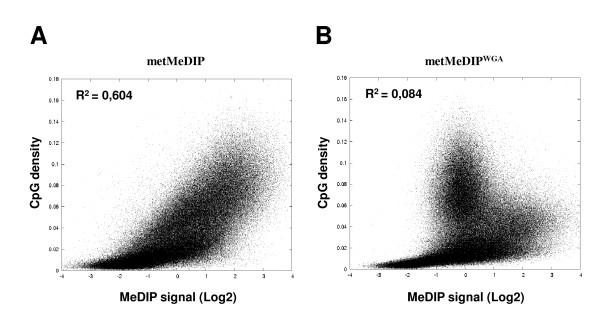
**Assessment of MeDIP efficiency using *in vitro *methylated DNA**. (A-B) Scatter plots of the metMeDIP (A) and metMeDIP^WGA ^(B) assays showing the enrichment levels (MeDIP signals) for all probes in the microarray relative to the CpG density in each individual probe surrounding region. R^2 ^= square Pearson correlation.

Next, we went on to test whether MeDIP signals accurately reflect methylation levels genome-wide. As M.SsssI treatment is expected to methylate every CpG in the starting DNA material, MeDIP enrichment is predicted in this context to directly reflect the density of CpG dinucleotides throughout the genome. The standard labeling protocol for microarray hybridization typically requires 2 μg of immunoprecipitated DNA (a yield that implies to start with large amounts, ~20 μg, of bulk genomic DNA). Therefore, we first pooled DNA samples that were obtained from 10 separate MeDIP experiments using *in vitro *methylated DNA (hereafter metMeDIP), and hybridized this pool to a custom-designed human promoter array (see Additional file 1 for the validation of a control set of gene promoters). For each probe, we compared the local CpG density (which integrates the contribution of CpGs surrounding each probe; see "Methods" section) to the resulting metMeDIP signal. In these conditions, we observed a marked correlation between the MeDIP signal and CpG density (Figure 2A; square Pearson correlation (R^2^) = 0,604), implying that the MeDIP assay accurately reflected the CpG density within each particular region. This further implied that the *in vitro *methylated DNA can be used to assess the efficiency of MeDIP assays also at the genome-wide scale, thus extending the observations from previous studies using arrays covering either a limited set of gene promoters [13] or a single chromosome [12]. In order to test whether the same results could be obtained using lower amounts of starting DNA, we amplified the DNA obtained from a single metMeDIP experiment using whole genome amplification or WGA (hereafter metMeDIP^WGA^), a procedure that has previously been used for the processing of MeDIP samples [15,16]. Surprisingly however, enrichment signals obtained from this metMeDIP^WGA ^experiment displayed lower correlation with the CpG density (Figure 2B; R^2 ^= 0,084). Notably, we observed a loss of correlation between metMeDIP^WGA ^signal and CpG content for regions sequences with relative CpG density higher than 0.04 (Figure 2B), indicating a strong amplification-induced bias for regions with high CpG content.

**Figure 3:**
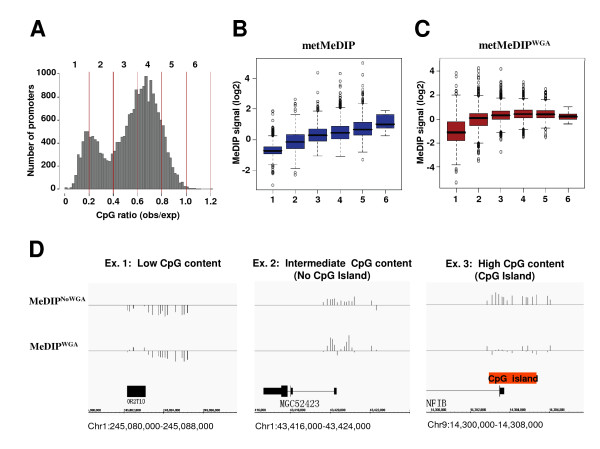
**WGA shows a bias against CpG-rich promoters**. (A) The histogram represents the distribution of observed *versus *expected CpG frequencies for all gene promoters analyzed. Six promoter populations were defined based on their CpG contents (vertical lines). (B-C) Box plots showing the MeDIP signals *per *promoter from metMeDIP (B) or metMeDIP^WGA ^(C) assays according to the promoter populations defined in (A). (D) Examples of methylation profiles for promoters with low, intermediate and high CpG contents. The presence of a CpG island in the gene promoter shown in example #3 is indicated.

The above results suggested an effect of WGA amplification on the signals observed at promoter regions that harbored high CpG content. To accurately assess the bias introduced by DNA amplification using WGA, we first classified the complete set of promoters covered by the microarray into six groups, based on their CpG contents [17] (Figure 3A). Next, we quantified the relative MeDIP signal *per *promoter by calculating the average enrichment signals for the probes covering each promoter region and compared the range of methylation signals for promoters exhibiting varying degrees of CpG content. As shown in figure 3B, the median methylation signals in metMeDIP assays increased proportionally with the frequency of CpGs. We observed a similar correlation with metMeDIP^WGA ^for the CpG-low and -intermediate promoters (CpG ratios between 0 and 0.6), though not for the CpG-high promoters (Figure 3C). Indeed, both CpG-low and CpG-intermediate promoters displayed similar methylation profiles in metMeDIP and metMeDIP^WGA ^experiments (Figure 3D, examples 1 and 2), whereas CpG-high promoters displayed high methylation signals in the metMeDIP experiment only (Figure 3D, example #3 of a CpG island containing promoter). Overall, these data demonstrated that MeDIP signals obtained from *in vitro *methylated DNA accurately correlate with the CpG content of gene promoters, highlighting its adequate use to quantify methylation levels. They also reveal a strong bias against CpG-rich promoters when treated by WGA, most probably due to the poor amplification of CG-rich sequences during the PCR step.

**Figure 4:**
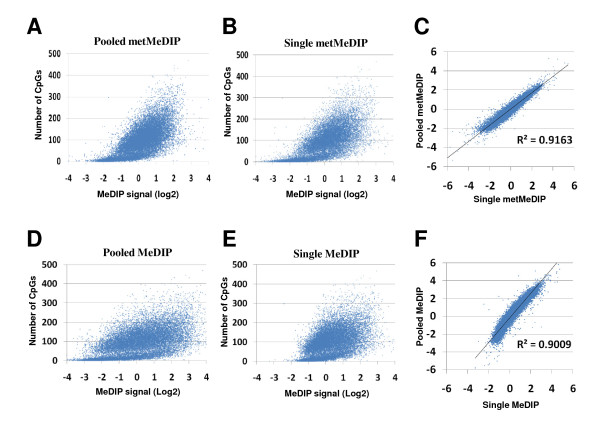
**Similar results are obtained from pooled or single MeDIP assays**. The average MeDIP signal *per *promoter obtained from pooled (A) or single (B) metMeDIP and pooled (D) or single (E) MeDIP assays were plotted against the CpG content of each promoter region. (C, F) Correlation between pooled and single metMeDIP (C) or MeDIP (F) experiments (R^2 ^= square Pearson correlation).

Finally, we asked whether we could improve the sensitivity of MeDIP assay in order to use reduced amount of starting DNA, while keeping with unbiased results. Initially, we repeated the assays using either different concentrations of the precipitating antibody or decreased numbers of WGA cycles, but did not observe significant improvement then (data not shown). Subsequently, by modifying the labeling conditions (for details, see "*Methods*" section and Additional file 2), we reproducibly obtained enough labeled material for array hybridization, even when starting from a single metMeDIP experiment. Strikingly, these "single metMeDIP"- based experiments yielded as good correlations between MeDIP signals and CpG contents at gene promoters as those observed previously when starting from metMeDIP DNA pools (see Figure 4A-C; R^2 ^= 0,9163). To demonstrate that this was also the case when using samples from M.SsssI untreated SIALL genomic DNA, we further labeled and hybridized the DNAs that were immunoprecipitated from either one or ten pooled MeDIP experiments. As shown in figure 4D-F, both types of starting MeDIP experiments yielded consistent and highly correlated results (R^2 ^= 0.9009), thus demonstrating that hybridization of MeDIP materials obtained from only 2 μg of starting genomic DNA does not in fact require prior amplification.

## Conclusion

We have used *in vitro *methylated DNA samples in an unbiased approach to assess the efficiency of the MeDIP procedure. Verifying genome-wide correlations between MeDIP signals from *in vitro *methylated DNA samples and actual CpG contents at gene promoters was important since *in vitro *methylated DNA was proposed to be used routinely to assess absolute methylation levels in MeDIP assays [e.g. [12,13]]. In this regard, a recently developed algorithm aimed at the estimation of methylation levels at individual promoters, has been implemented to also take into account the signals from *in vitro *methylated DNA samples [12]. Recent works using single-base resolution maps of methylated cytosines in human embryonic stem cells have identified cytosine methylation in a non-CpG context [6,18]. Because MeDIP apparently enables the capture of non-CpG methylation [19], experimental and *in silico *attempts to quantify absolute levels of DNA methylation should also take this particular feature into account. While our results reveal the loss of signal for CpG-rich regions following WGA, quantitative information can still be retrieved for low and intermediate CpG promoters (Figure 3). The latter observation is particularly relevant as it is currently thought that these regions precisely undergo *de novo *methylation in transformed cells [e.g., [20]]. These points need to be considered when only a limited amount of DNA is available (e.g., in analysis of tumor tissues or rare cell subpopulations) and amplification-based methods such as WGA are unavoidable. Notice that the bias observed after WGA amplification in this study of DNA methylation may be of a broader interest, as this amplification procedure is commonly used in several other types of quantitative assays, including ChIP-on-chip, comparative genomic hybridization (CGH) and single nucleotide polymorphism (SNP) [21-24]. Finally, we also demonstrate here that consistent and accurate genome-wide methylation data can be reproducibly attained in array hybridization using MeDIP materials obtained from as little as 2 μg of starting genomic DNA (an amount that is regularly available from cancer tissue biopsies), without the need for additional amplification steps.

## Methods

### DNA preparation and in vitro methylation

Genomic DNA from the human T-acute lymphoblastic leukemia cell line SilALL [14] was sonicated to a range of 300-500 bp, using a Bioruptor (Diagenode, Liège, Belgium). *In vitro *methylation was achieved by incubating the sonicated DNA with 20 units of the CpG-methyltransferase M.SssI (New England Biolabs, Frankfurt, Germany) for 4 h at 37°C [25], followed by DNA purification with the Qiaquick PCR kit (Qiagen, Hilden, Germany).

### Methylated DNA Immunoprecipitation (MeDIP) assays

Methylated DNA was immunoprecipitated as described previously [8] with a few modifications. Briefly, 2 μg of denatured DNA was incubated with 2 μg of anti-5-methylcytidine antibody (Eurogentec, Seraing, Belgium) in IP buffer (10 mM Na-Phosphate pH 7.0, 0.14 M NaCl, 0.05% Triton X-100) for 2 h at 4°C. Antibody-bound DNA was collected with 40 μl of Dynabeads M-280 sheep anti-mouse IgG (Invitrogen Dynal, Oslo, Norway) for 1 h at 4°C on a rotating wheel and successively washed with buffer I (0.1% SDS, 1% Triton X-100, 2 mM EDTA, 20 mM Tris-HCl pH 8.1, 150 mM NaCl), buffer I complemented with 500 mM NaCl, LiCl buffer (250 mM LiCl, 1% IGEPAL-CA630, 1% deoxycholic acid, 1 mM EDTA, 10 mM Tris-HCl pH 8.1) and twice with TE (10 mM Tris·Cl, 1 mM EDTA pH 8.0). The beads were resuspended in 125 μl PK buffer (50 mM Tris pH 8.0, 10 mM EDTA, 0.5% SDS, 35 μg proteinase K) and incubated for 3 h at 50°C. DNA was extracted by standard phenol/chloroform procedure and purified as above. The DNA from one MeDIP experiment was subjected to amplification using the WGA-2 kit (Sigma-Aldrich, Taufkirchen, Germany). Subsequently, 2 μg of DNA from either 10 pooled samples or a WGA amplified sample (MeDIP^WGA^), along with their corresponding whole genomic DNA (input), were labeled using the BioPrime Array CGH Genomic Labeling System (Invitrogen) and hybridized to a custom human promoter array (Agilent, Santa Clara, USA) containing 236,992 probes, following the microarray manufacturer's instructions. In experiments shown in Figure 3, the DNA obtained from a single MeDIP experiment (usually ~250 ng) was labeled using a modified protocol in which Cy3-dUTP and Cy5-dUTP were replaced by Cy3-dCTP and Cy5-dCTP during the labeling procedure. In these conditions, we generally obtained more than 4 μg of efficiently labeled DNA (Note that, when using the classical labeling conditions and 2 μg of starting DNA material, we did not obtain labeled samples of high-enough quality to be hybridized onto Agilent arrays; Additional file 2). Finally, median-normalized log_2 _enrichment ratios (Medip/Input) were calculated using CoCAS software [26]. Experiments were performed in duplicate and showed very high correlation in all cases (R^2 ^> 0.93).

### CpG density

To integrate the contributions of CpG dinucleotides around each probe to the MeDIP signal, we calculated a local CpG density. To compute CpG densities, we weighted the CpGs found in the 800 bp genomic region surrounding each probe by their distance to the probe using a Gaussian distribution.

## List of abbreviations used

MeDIP: Methylated DNA Immunoprecipitation; WGA: Whole Genome Amplification; CGH: comparative genomic hybridization; SNP: single-nucleotide polymorphism.

## Competing interests

The authors declare that they have no competing interests.

## Authors' contributions

JJ and AP carried out MeDIP experiments. TB designed the human promoter array and developed bioinformatic tools. JJ, AP, SJ and SS analyzed the data. JJ, AP, SS and PF wrote the manuscript. All authors read and approved the final manuscript

## Supplementary Material

Additional file 1 **Confirmation of methylation results obtained by qPCR**. Methylation profiles obtained in pooled metMeDIP or MeDIP hybridization experiments for gene promoters tested by qPCR in Figure 1 are shown. Click here for file

Additional file 2 **Assessment of the efficiency of the labeling procedure**. Control DNA and MeDIP samples were labeled with either Cy5-dUTP or Cy5-dCTP and the labeling efficiency was compared. Click here for file
