# Supplementary material for: Assessing the efficiency and significance of Methylated DNA Immunoprecipitation (MeDIP) assays in using in vitro methylated genomic DNA
Source: BMC Res Notes. 2010 Sep 16;3:240. doi: 10.1186/1756-0500-3-240 (PMC2949662; doi:10.1186/1756-0500-3-240)
Supplement: Additional file 1 — Confirmation of methylation results obtained by qPCR. Methylation profiles obtained in pooled metMeDIP or MeDIP hybridization experiments for gene promoters tested by qPCR in Figure 1 are shown. [file 1756-0500-3-240-S1.PPT]

## Slide 1
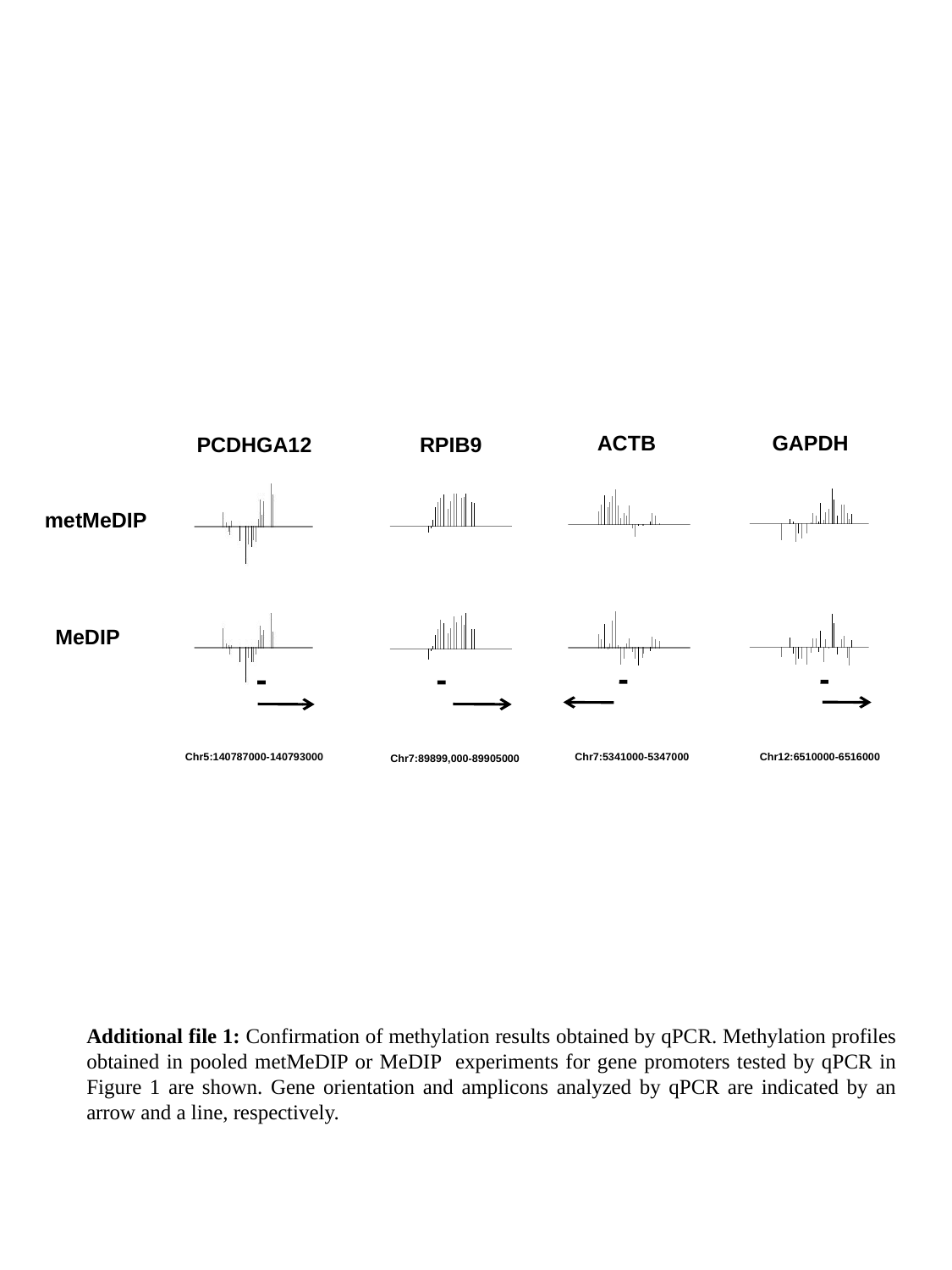

ACTB
GAPDH
PCDHGA12
RPIB9
metMeDIP
MeDIP
Chr5:140787000-140793000
Chr7:5341000-5347000
Chr12:6510000-6516000
Chr7:89899,000-89905000
Additional file 1: Confirmation of methylation results obtained by qPCR. Methylation profiles obtained in pooled metMeDIP or MeDIP experiments for gene promoters tested by qPCR in Figure 1 are shown. Gene orientation and amplicons analyzed by qPCR are indicated by an arrow and a line, respectively.
