# Supplementary material for: Assessing the efficiency and significance of Methylated DNA Immunoprecipitation (MeDIP) assays in using in vitro methylated genomic DNA
Source: BMC Res Notes. 2010 Sep 16;3:240. doi: 10.1186/1756-0500-3-240 (PMC2949662; doi:10.1186/1756-0500-3-240)
Supplement: Additional file 2 — Assessment of the efficiency of the labeling procedure. Control DNA and MeDIP samples were labeled with either Cy5-dUTP or Cy5-dCTP and the labeling efficiency was compared. [file 1756-0500-3-240-S2.PPT]

## Slide 1
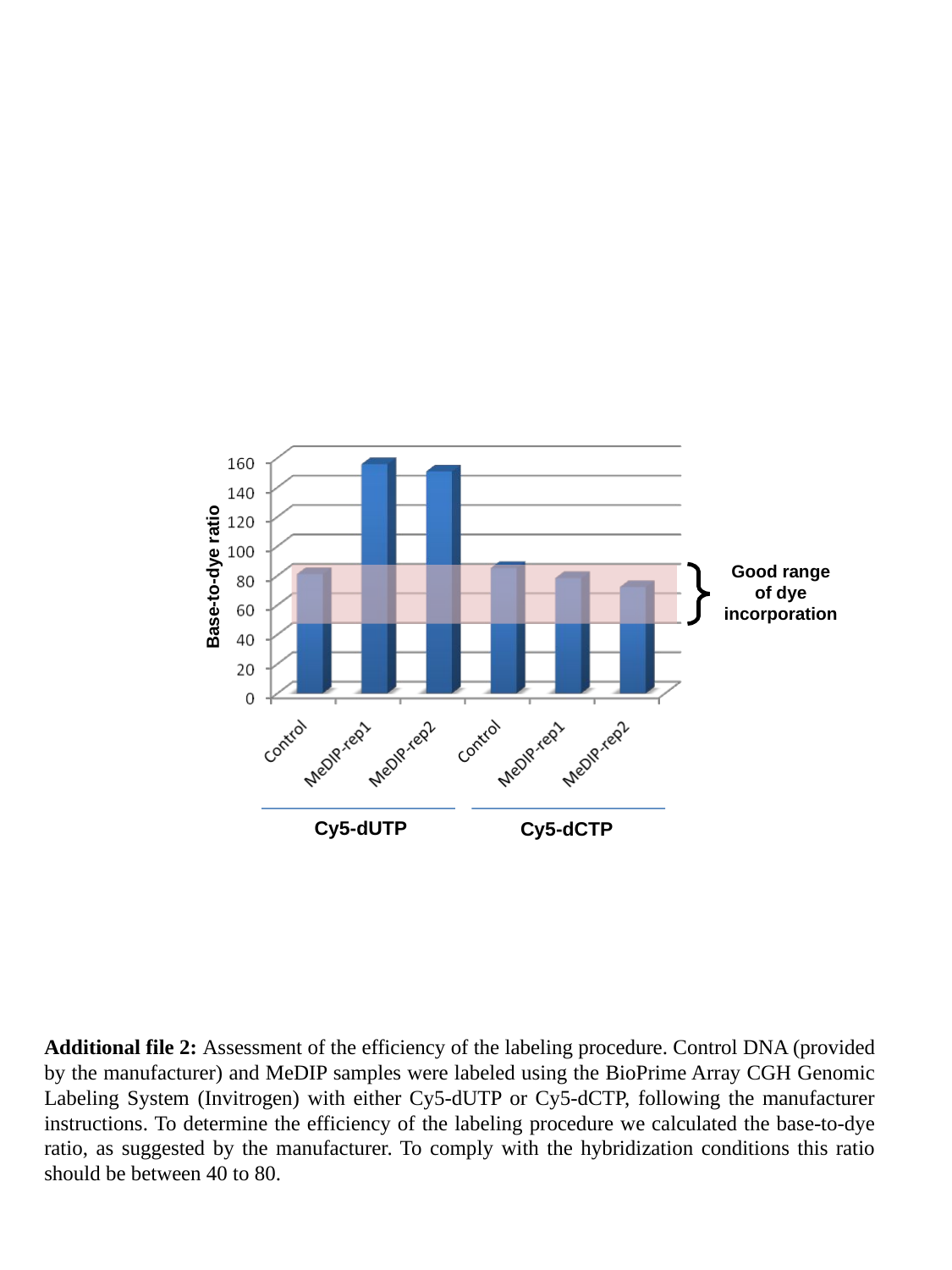

Good range
of dye incorporation
Base-to-dye ratio
Cy5-dUTP
Cy5-dCTP
Additional file 2: Assessment of the efficiency of the labeling procedure. Control DNA (provided by the manufacturer) and MeDIP samples were labeled using the BioPrime Array CGH Genomic Labeling System (Invitrogen) with either Cy5-dUTP or Cy5-dCTP, following the manufacturer instructions. To determine the efficiency of the labeling procedure we calculated the base-to-dye ratio, as suggested by the manufacturer. To comply with the hybridization conditions this ratio should be between 40 to 80.
